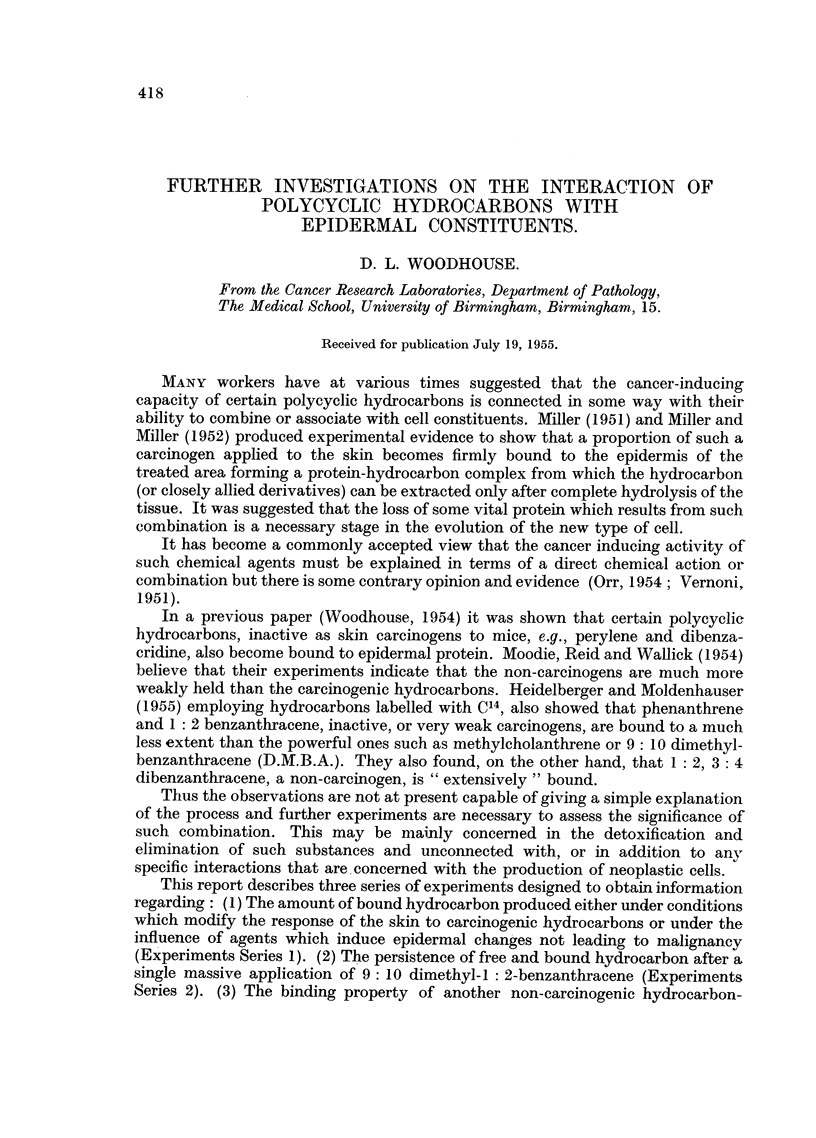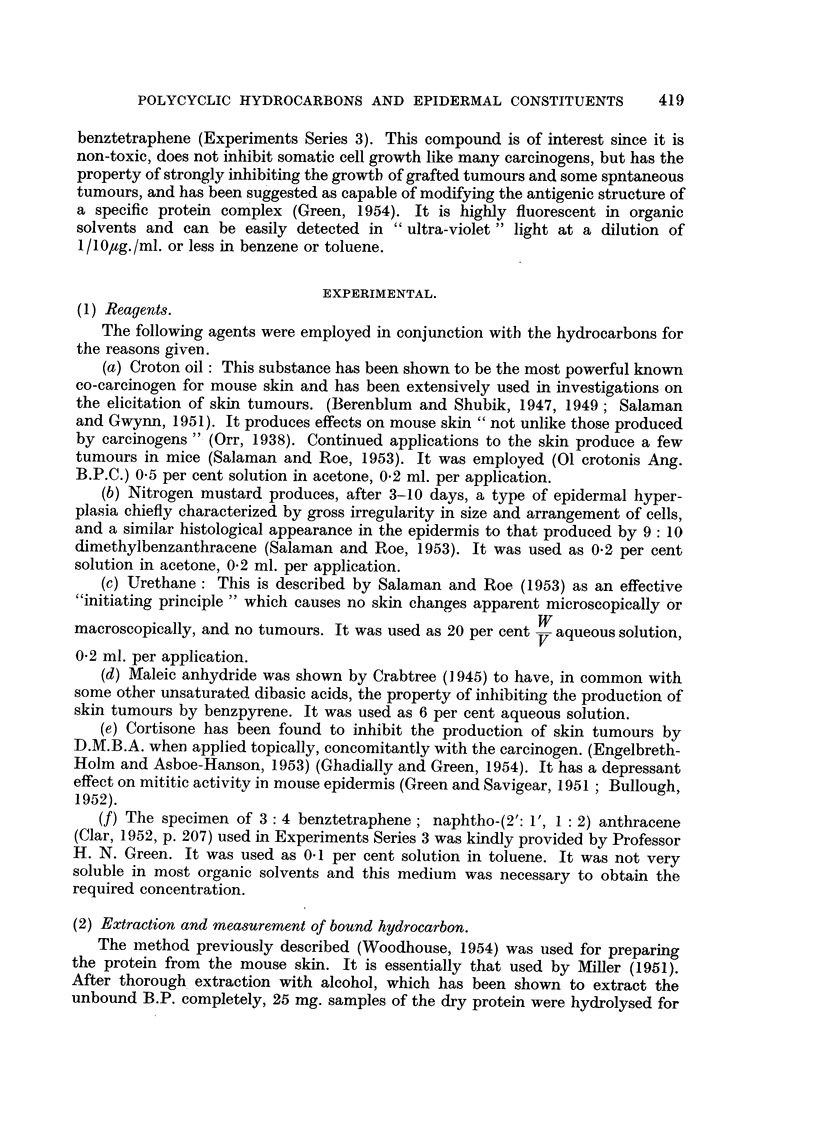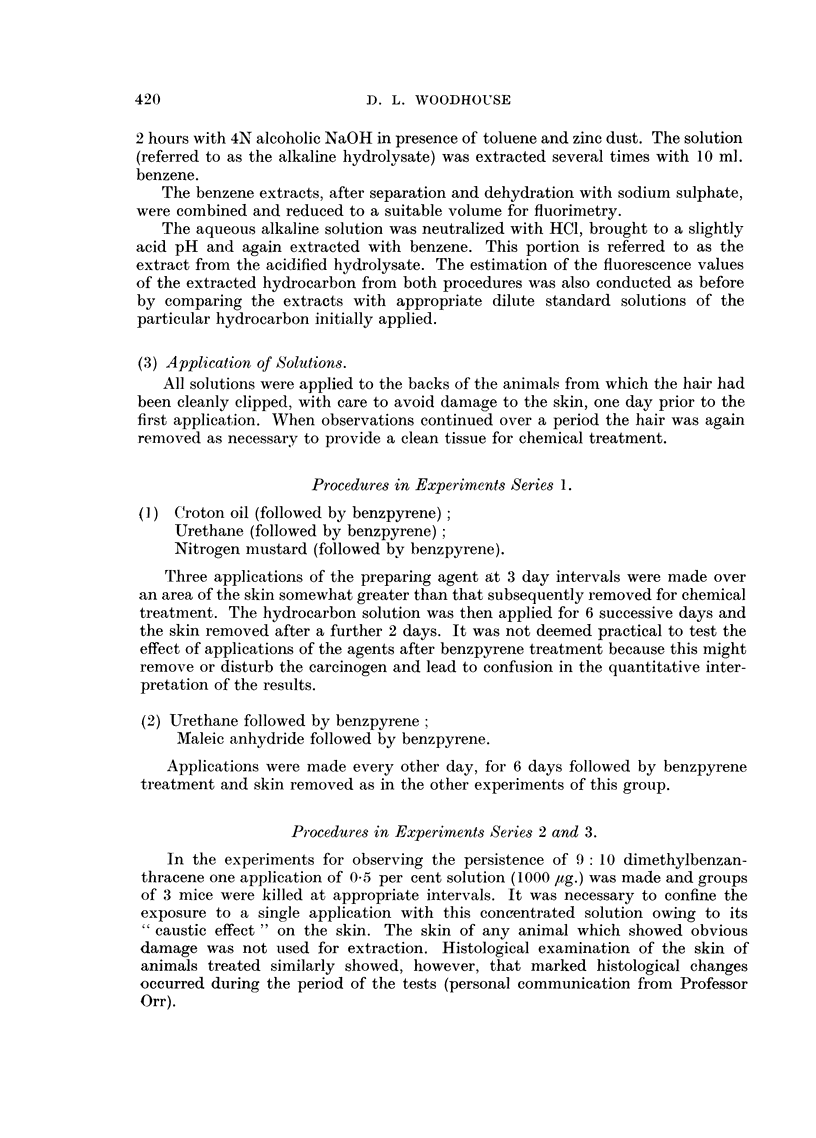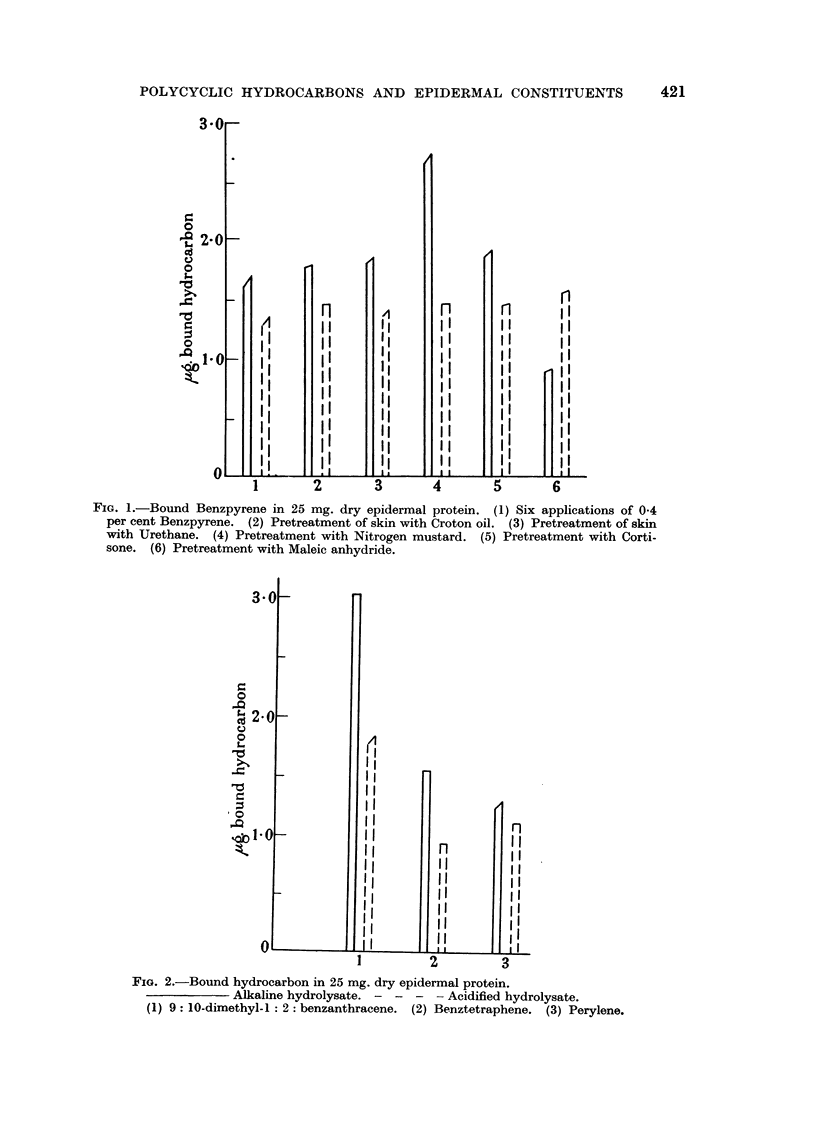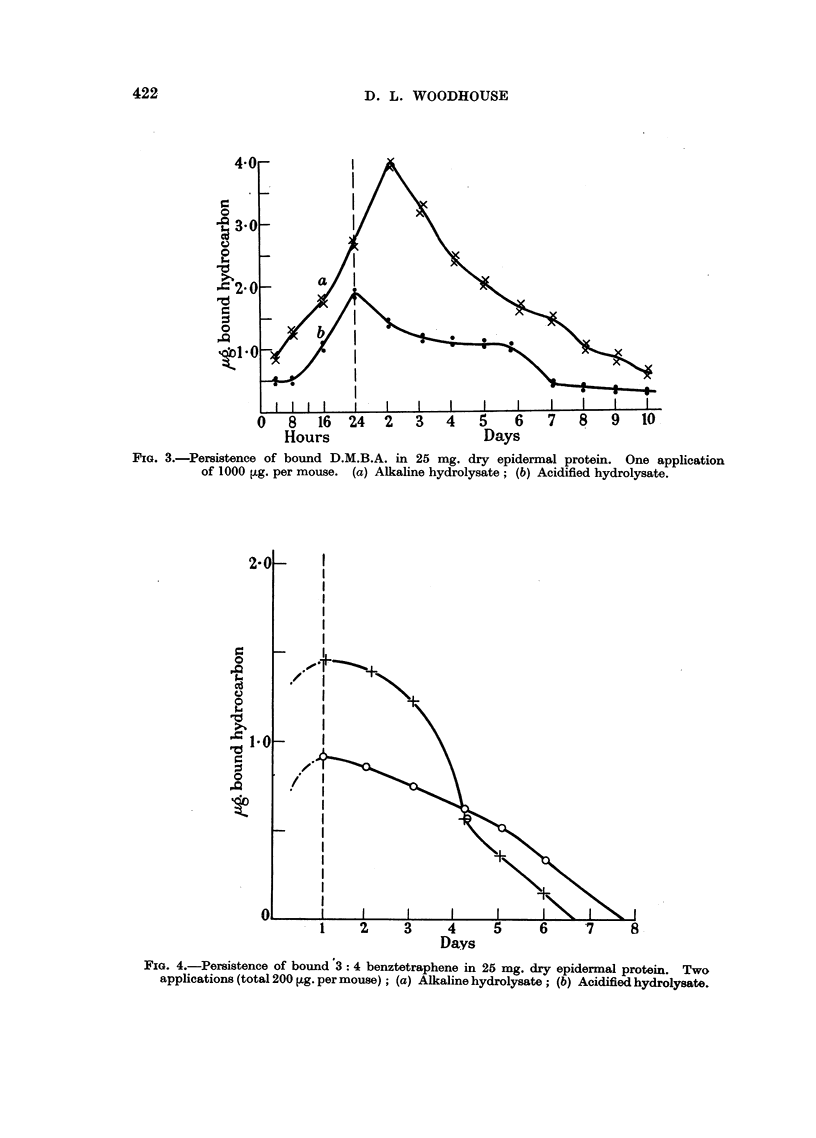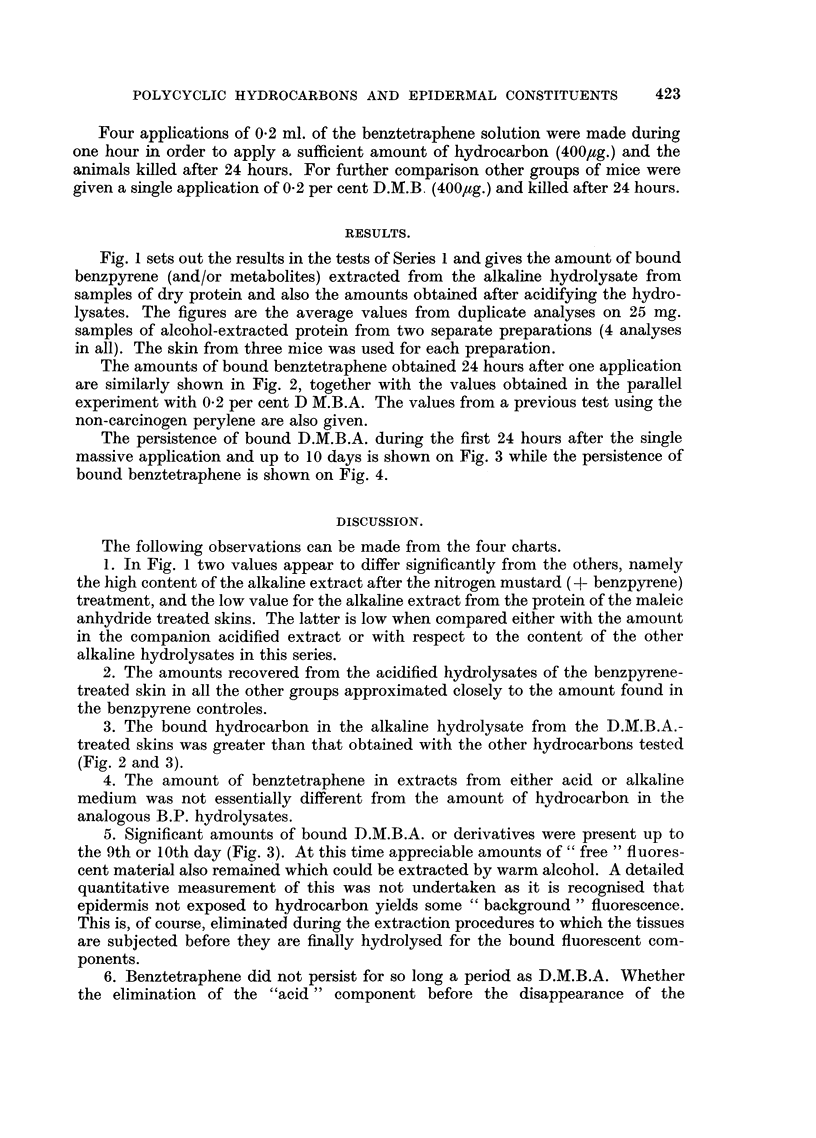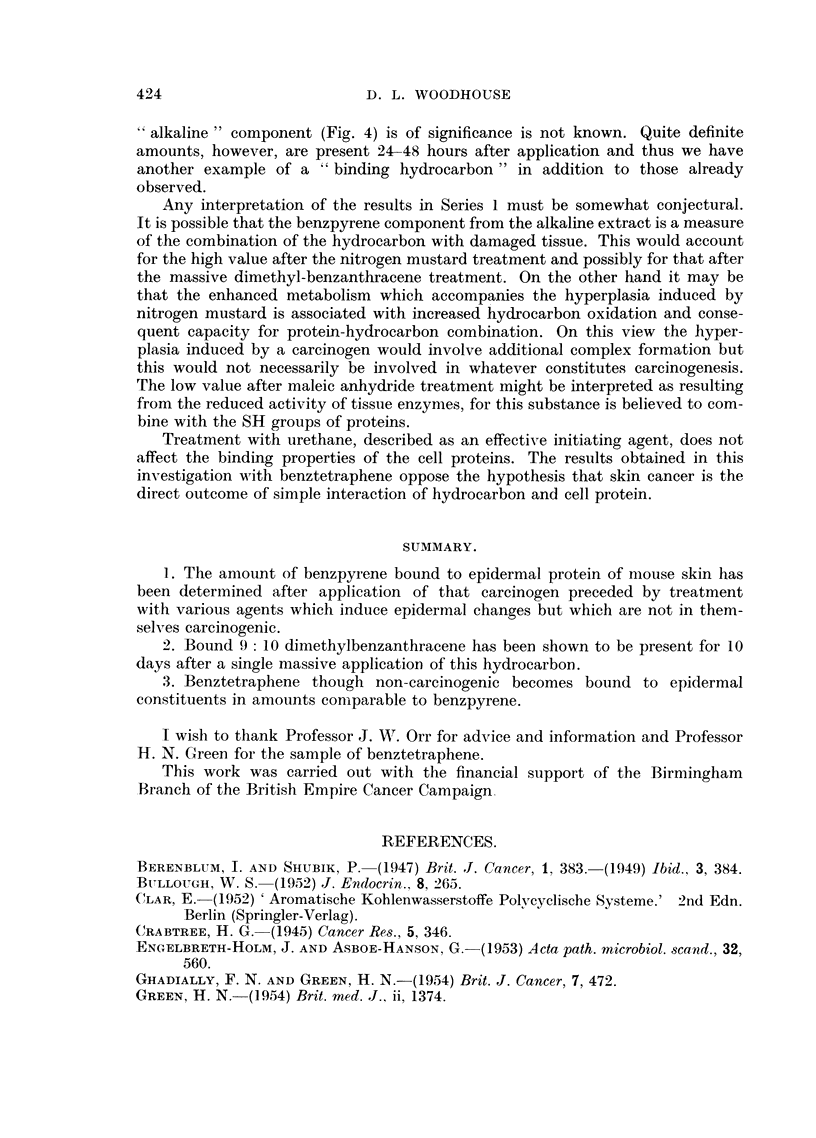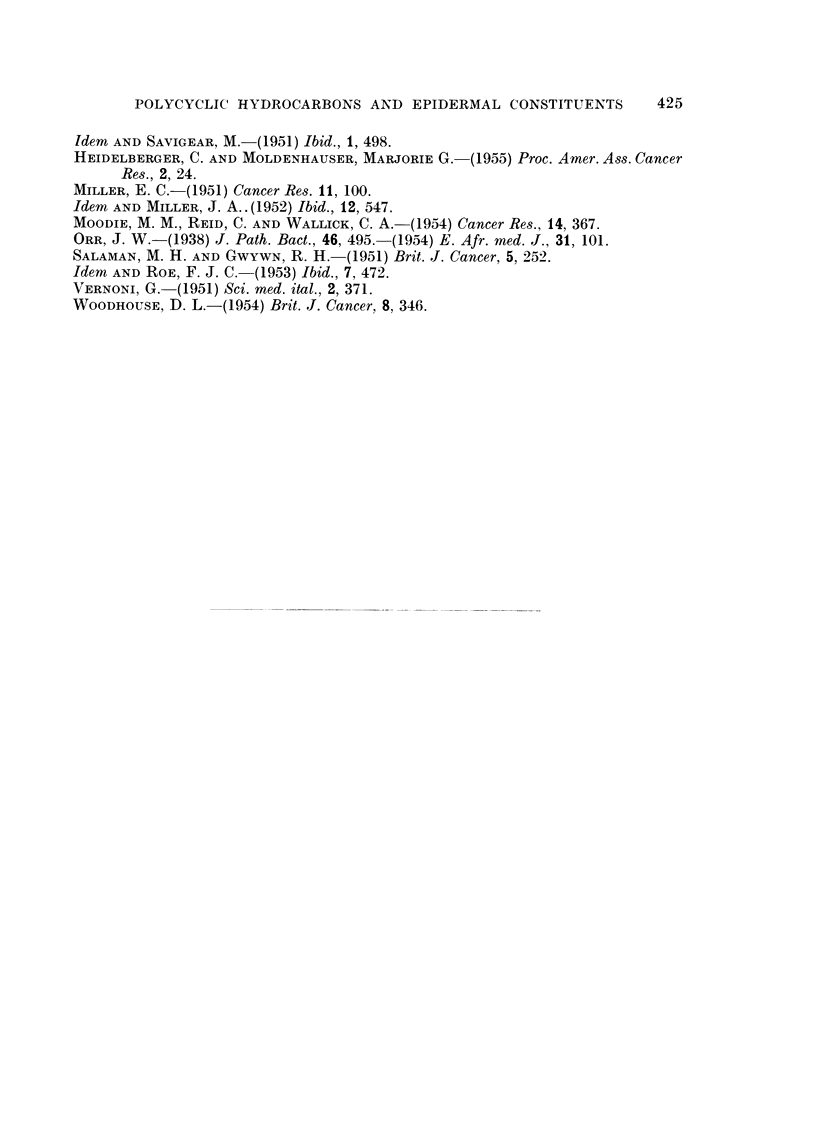# Further Investigations on the Interaction of Polycyclic Hydrocarbons with Epidermal Constituents

**DOI:** 10.1038/bjc.1955.40

**Published:** 1955-09

**Authors:** D. L. Woodhouse


### FURTHER INVESTIGATIONS ON THE INTERACTION OF POLYCYCLIC HYDROCARBONS WITH EPIDERMAL CONSTITUENTS.

D. L. WOODHOUSE.

*From the Cancer Research Laboratories, Department of Pathology, The Medical School, University of Birmingham, Birmingham, 15.*

Received for publication July 19, 1955.

MANY workers have at various times suggested that the cancer-inducing capacity of certain polycyclic hydrocarbons is connected in some way with their ability to combine or associate with cell constituents. Miller (1951) and Miller and Miller (1952) produced experimental evidence to show that a proportion of such a carcinogen applied to the skin becomes firmly bound to the epidermis of the treated area forming a protein-hydrocarbon complex from which the hydrocarbon (or closely allied derivatives) can be extracted only after complete hydrolysis of the tissue. It was suggested that the loss of some vital protein which results from such combination is a necessary stage in the evolution of the new type of cell.

It has become a commonly accepted view that the cancer inducing activity of such chemical agents must be explained in terms of a direct chemical action or combination but there is some contrary opinion and evidence (Orr, 1954 ; Vernoni, 1951).

In a previous paper (Woodhouse, 1954) it was shown that certain polycyclic hydrocarbons, inactive as skin carcinogens to mice, *e.g.*, perylene and dibenzacridine, also become bound to epidermal protein. Moodie, Reid and Wallick (1954) believe that their experiments indicate that the non-carcinogens are much more weakly held than the carcinogenic hydrocarbons. Heidelberger and Moldenhauser (1955) employing hydrocarbons labelled with $C^{14}$, also showed that phenanthrene and 1 : 2 benzanthracene, inactive, or very weak carcinogens, are bound to a much less extent than the powerful ones such as methylcholanthrene or 9 : 10 dimethylbenzanthracene (D.M.B.A.). They also found, on the other hand, that 1 : 2, 3 : 4 dibenzanthracene, a non-carcinogen, is " extensively " bound.

Thus the observations are not at present capable of giving a simple explanation of the process and further experiments are necessary to assess the significance of such combination. This may be mainly concerned in the detoxification and elimination of such substances and unconnected with, or in addition to any specific interactions that are concerned with the production of neoplastic cells.

This report describes three series of experiments designed to obtain information regarding : (1) The amount of bound hydrocarbon produced either under conditions which modify the response of the skin to carcinogenic hydrocarbons or under the influence of agents which induce epidermal changes not leading to malignancy (Experiments Series 1). (2) The persistence of free and bound hydrocarbon after a single massive application of 9 : 10 dimethyl-1 : 2-benzanthracene (Experiments Series 2). (3) The binding property of another non-carcinogenic hydrocarbonbenztetraphene (Experiments Series 3). This compound is of interest since it is non-toxic, does not inhibit somatic cell growth like many carcinogens, but has the property of strongly inhibiting the growtb of grafted tumours and some spntaneous tumours, and has been suggested as capable of modifying the antigenic structure of a specific protein complex (Green, 1954). It is highly fluorescent in organic solvents and can be easily detected in " ultra-violet " light at a dilution of $1/10\mu g./ml.$ or less in benzene or toluene.

### EXPERIMENTAL.

### (1) *Reagents.*

The following agents were employed in conjunction witb the hydrocarbons for the reasons given.

(*a*) Croton oil : This substance has been shown to be the most powerful known co-carcinogen for mouse skin and has been extensively used in investigations on the elicitation of skin tumours. (Berenblum and Shubik, 1947, 1949 ; Salaman and Gwynn, 1951). It produces effects on mouse skin " not unlike those produced by carcinogens " (Orr, 1938). Continued applications to the skin produce a few tumours in mice (Salaman and Roe, 1953). It was employed (Ol crotonis Ang. B.P.C.) 0·5 per cent solution in acetone, 0·2 ml. per application.

(*b*) Nitrogen mustard produces, after 3–10 days, a type of epidermal hyperplasia chiefly characterized by gross irregularity in size and arrangement of cells, and a similar histological appearance in the epidermis to that produced by 9 : 10 dimethylbenzanthracene (Salaman and Roe, 1953). It was used as 0·2 per cent solution in acetone, 0·2 ml. per application.

(*c*) Urethane : This is described by Salaman and Roe (1953) as an effective "initiating principle " which causes no skin changes apparent microscopically or macroscopically, and no tumours. It was used as 20 per cent $\frac{W}{V}$ aqueous solution, 0·2 ml. per application.

(*d*) Maleic anhydride was shown by Crabtree (1945) to have, in common with some other unsaturated dibasic acids, the property of inhibiting the production of skin tumours by benzpyrene. It was used as 6 per cent aqueous solution.

(*e*) Cortisone has been found to inhibit the production of skin tumours by D.M.B.A. when applied topically, concomitantly with the carcinogen. (Engelbreth-Holm and Asboe-Hanson, 1953) (Ghadially and Green, 1954). It has a depressant effect on mititic activity in mouse epidermis (Green and Savigear, 1951 ; Bullough, 1952).

(*f*) The specimen of 3 : 4 benztetraphene ; naphtho-(2′: 1′, 1 : 2) anthracene (Clar, 1952, p. 207) used in Experiments Series 3 was kindly provided by Professor H. N. Green. It was used as 0·1 per cent solution in toluene. It was not very soluble in most organic solvents and this medium was necessary to obtain the required concentration.

### (2) *Extraction and measurement of bound hydrocarbon.*

The method previously described (Woodhouse, 1954) was used for preparing the protein from the mouse skin. It is essentially that used by Miller (1951). After thorough extraction with alcohol, which has been shown to extract the unbound B.P. completely, 25 mg. samples of the dry protein were hydrolysed for

2 hours with 4N alcoholic NaOH in presence of toluene and zinc dust. The solution (referred to as the alkaline hydrolysate) was extracted several times with 10 ml. benzene.

The benzene extracts, after separation and dehydration with sodium sulphate, were combined and reduced to a suitable volume for fluorimetry.

The aqueous alkaline solution was neutralized with HCl, brought to a slightly acid pH and again extracted with benzene. This portion is referred to as the extract from the acidified hydrolysate. The estimation of the fluorescence values of the extracted hydrocarbon from both procedures was also conducted as before by comparing the extracts with appropriate dilute standard solutions of the particular hydrocarbon initially applied.

(3) *Application of Solutions.*

All solutions were applied to the backs of the animals from which the hair had been cleanly clipped, with care to avoid damage to the skin, one day prior to the first application. When observations continued over a period the hair was again removed as necessary to provide a clean tissue for chemical treatment.

### *Procedures in Experiments Series* 1.

(1) Croton oil (followed by benzpyrene) ;
Urethane (followed by benzpyrene) ;
Nitrogen mustard (followed by benzpyrene).

Three applications of the preparing agent at 3 day intervals were made over an area of the skin somewhat greater than that subsequently removed for chemical treatment. The hydrocarbon solution was then applied for 6 successive days and the skin removed after a further 2 days. It was not deemed practical to test the effect of applications of the agents after benzpyrene treatment because this might remove or disturb the carcinogen and lead to confusion in the quantitative interpretation of the results.

(2) Urethane followed by benzpyrene ;
Maleic anhydride followed by benzpyrene.

Applications were made every other day, for 6 days followed by benzpyrene treatment and skin removed as in the other experiments of this group.

### *Procedures in Experiments Series* 2 *and* 3.

In the experiments for observing the persistence of 9 : 10 dimethylbenzanthracene one application of 0·5 per cent solution (1000 $\mu$g.) was made and groups of 3 mice were killed at appropriate intervals. It was necessary to confine the exposure to a single application with this concentrated solution owing to its "caustic effect" on the skin. The skin of any animal which showed obvious damage was not used for extraction. Histological examination of the skin of animals treated similarly showed, however, that marked histological changes occurred during the period of the tests (personal communication from Professor Orr).

Fig. 1.—Bound Benzpyrene in 25 mg. dry epidermal protein. (1) Six applications of 0·4 per cent Benzpyrene. (2) Pretreatment of skin with Croton oil. (3) Pretreatment of skin with Urethane. (4) Pretreatment with Nitrogen mustard. (5) Pretreatment with Cortisone. (6) Pretreatment with Maleic anhydride.

Fig. 2.—Bound hydrocarbon in 25 mg. dry epidermal protein.
———— Alkaline hydrolysate. – – – – Acidified hydrolysate.
(1) 9 : 10-dimethyl-1 : 2 : benzanthracene. (2) Benztetraphene. (3) Perylene.

Fig. 3.—Persistence of bound D.M.B.A. in 25 mg. dry epidermal protein. One application of 1000 μg. per mouse. (*a*) Alkaline hydrolysate ; (*b*) Acidified hydrolysate.

Fig. 4.—Persistence of bound 3 : 4 benztetraphene in 25 mg. dry epidermal protein. Two applications (total 200 μg. per mouse) ; (*a*) Alkaline hydrolysate ; (*b*) Acidified hydrolysate.

Four applications of 0·2 ml. of the benztetraphene solution were made during one hour in order to apply a sufficient amount of hydrocarbon (400$\mu$g.) and the animals killed after 24 hours. For further comparison other groups of mice were given a single application of 0·2 per cent D.M.B. (400$\mu$g.) and killed after 24 hours.

### RESULTS.

Fig. 1 sets out the results in the tests of Series 1 and gives the amount of bound benzpyrene (and/or metabolites) extracted from the alkaline hydrolysate from samples of dry protein and also the amounts obtained after acidifying the hydrolysates. The figures are the average values from duplicate analyses on 25 mg. samples of alcohol-extracted protein from two separate preparations (4 analyses in all). The skin from three mice was used for each preparation.

The amounts of bound benztetraphene obtained 24 hours after one application are similarly shown in Fig. 2, together with the values obtained in the parallel experiment with 0·2 per cent D M.B.A. The values from a previous test using the non-carcinogen perylene are also given.

The persistence of bound D.M.B.A. during the first 24 hours after the single massive application and up to 10 days is shown on Fig. 3 while the persistence of bound benztetraphene is shown on Fig. 4.

### DISCUSSION.

The following observations can be made from the four charts.

1. In Fig. 1 two values appear to differ significantly from the others, namely the high content of the alkaline extract after the nitrogen mustard (+ benzpyrene) treatment, and the low value for the alkaline extract from the protein of the maleic anhydride treated skins. The latter is low when compared either with the amount in the companion acidified extract or with respect to the content of the other alkaline hydrolysates in this series.
2. The amounts recovered from the acidified hydrolysates of the benzpyrene-treated skin in all the other groups approximated closely to the amount found in the benzpyrene controles.
3. The bound hydrocarbon in the alkaline hydrolysate from the D.M.B.A.-treated skins was greater than that obtained with the other hydrocarbons tested (Fig. 2 and 3).
4. The amount of benztetraphene in extracts from either acid or alkaline medium was not essentially different from the amount of hydrocarbon in the analogous B.P. hydrolysates.
5. Significant amounts of bound D.M.B.A. or derivatives were present up to the 9th or 10th day (Fig. 3). At this time appreciable amounts of " free " fluorescent material also remained which could be extracted by warm alcohol. A detailed quantitative measurement of this was not undertaken as it is recognised that epidermis not exposed to hydrocarbon yields some " background " fluorescence. This is, of course, eliminated during the extraction procedures to which the tissues are subjected before they are finally hydrolysed for the bound fluorescent components.
6. Benztetraphene did not persist for so long a period as D.M.B.A. Whether the elimination of the "acid" component before the disappearance of the

"alkaline" component (Fig. 4) is of significance is not known. Quite definite amounts, however, are present 24–48 hours after application and thus we have another example of a "binding hydrocarbon" in addition to those already observed.

Any interpretation of the results in Series 1 must be somewhat conjectural. It is possible that the benzpyrene component from the alkaline extract is a measure of the combination of the hydrocarbon with damaged tissue. This would account for the high value after the nitrogen mustard treatment and possibly for that after the massive dimethyl-benzanthracene treatment. On the other hand it may be that the enhanced metabolism which accompanies the hyperplasia induced by nitrogen mustard is associated with increased hydrocarbon oxidation and consequent capacity for protein-hydrocarbon combination. On this view the hyperplasia induced by a carcinogen would involve additional complex formation but this would not necessarily be involved in whatever constitutes carcinogenesis. The low value after maleic anhydride treatment might be interpreted as resulting from the reduced activity of tissue enzymes, for this substance is believed to combine with the SH groups of proteins.

Treatment with urethane, described as an effective initiating agent, does not affect the binding properties of the cell proteins. The results obtained in this investigation with benztetraphene oppose the hypothesis that skin cancer is the direct outcome of simple interaction of hydrocarbon and cell protein.

### SUMMARY.

1. The amount of benzpyrene bound to epidermal protein of mouse skin has been determined after application of that carcinogen preceded by treatment with various agents which induce epidermal changes but which are not in themselves carcinogenic.

2. Bound 9 : 10 dimethylbenzanthracene has been shown to be present for 10 days after a single massive application of this hydrocarbon.

3. Benztetraphene though non-carcinogenic becomes bound to epidermal constituents in amounts comparable to benzpyrene.

I wish to thank Professor J. W. Orr for advice and information and Professor H. N. Green for the sample of benztetraphene.

This work was carried out with the financial support of the Birmingham Branch of the British Empire Cancer Campaign.

### REFERENCES.

Berenblum, I. and Shubik, P.—(1947) *Brit. J. Cancer*, **1**, 383.—(1949) *Ibid.*, **3**, 384.

Bullough, W. S.—(1952) *J. Endocrin.*, **8**, 265.

Clar, E.—(1952) 'Aromatische Kohlenwasserstoffe Polycyclische Systeme.' 2nd Edn. Berlin (Springler-Verlag).

Crabtree, H. G.—(1945) *Cancer Res.*, **5**, 346.

Engelbreth-Holm, J. and Asboe-Hanson, G.—(1953) *Acta path. microbiol. scand.*, **32**, 560.

Ghadially, F. N. and Green, H. N.—(1954) *Brit. J. Cancer*, **7**, 472.

Green, H. N.—(1954) *Brit. med. J.*, ii, 1374.

*Idem* AND SAVIGEAR, M.—(1951) *Ibid.*, **1**, 498.
HEIDELBERGER, C. AND MOLDENHAUSER, MARJORIE G.—(1955) *Proc. Amer. Ass. Cancer Res.*, **2**, 24.
MILLER, E. C.—(1951) *Cancer Res.* **11**, 100.
*Idem* AND MILLER, J. A..(1952) *Ibid.*, **12**, 547.
MOODIE, M. M., REID, C. AND WALLICK, C. A.—(1954) *Cancer Res.*, **14**, 367.
ORR, J. W.—(1938) *J. Path. Bact.*, **46**, 495.—(1954) *E. Afr. med. J.*, **31**, 101.
SALAMAN, M. H. AND GWYWN, R. H.—(1951) *Brit. J. Cancer*, **5**, 252.
*Idem* AND ROE, F. J. C.—(1953) *Ibid.*, **7**, 472.
VERNONI, G.—(1951) *Sci. med. ital.*, **2**, 371.
WOODHOUSE, D. L.—(1954) *Brit. J. Cancer*, **8**, 346.